# Integrating avatar technology into a telemedicine application in heart failure patients: A pilot study

**DOI:** 10.1007/s00508-022-02150-8

**Published:** 2023-02-02

**Authors:** Arno Joachim Gingele, Hesam Amin, Aniek Vaassen, Ivana Schnur, Cathy Pearl, Hans-Peter Brunner-La Rocca, Josiane Boyne

**Affiliations:** 1 Department of Cardiology, Maastricht University Medical Centre, P.Debyelaan 25, 6229 HX Maastricht, The Netherlands; 2 Sananet Care BV, Sittard, The Netherlands; 3 Sense.ly company, San Francisco, USA

**Keywords:** Health care, eHealth, Telemedicine, Cardiovascular nursing, Self-care

## Abstract

**Background:**

Heart failure is a severe condition and telemedicine can improve the care of heart failure. Many patients are unable to use telemedicine applications due to visual impairment and limited health-related literacy. Avatar technology might help to overcome these limitations.

**Methods:**

A telemedicine application was combined with a nurse avatar and offered to heart failure outpatients for 3 months. System usability and patient satisfaction were evaluated monthly by the system usability score (maximum score=100) and the patient satisfaction scale (maximum score=50).

**Results:**

In total, 37 heart failure patients were enrolled. The mean system usability score after 1 month was 73 (standard deviation=24) and 72 (standard deviation=10) after 3 months of follow-up, which was not significantly different (*p* = 0.40). The mean patient satisfaction scale after 1 month was 42 (standard deviation=5) and 39 (standard deviation=8) after 3 months, which was not significantly different (*p* = 0.10).

**Conclusion:**

A nurse look-a-like avatar integrated into a telemedicine application was positively assessed by heart failure patients. Future studies are warranted to clarify the role of avatar technology in telemedicine.

**Supplementary Information:**

The online version of this article (10.1007/s00508-022-02150-8) contains supplementary material, which is available to authorized users.

## Introduction

Heart failure (HF) is a severe syndrome with high morbidity and mortality [1]. It affects 1–3% of the general population in western countries and nearly 10% of the very old (≥ 80 years of age) [2]. Healthcare consumption of HF patients is extensive, resulting in a significant burden on national healthcare systems [3]. Consequently, redesigning healthcare is urgently needed [4].

Telemedicine, the use of technology to facilitate communication between patients and healthcare professionals, can play an important role in the reorganization of HF care as it allows remote monitoring and education of patients [5]. Telemedicine has the potential to reduce healthcare utilization, decreasing mortality and HF hospitalizations [6], and increasing disease-specific knowledge and self-care in HF patients [7]. The HF coach is a telemedicine application that regularly provides health questionnaires as well as educational sessions to HF outpatients in order to prevent clinical deterioration and improve disease-specific knowledge [8].

Nevertheless, many patients are not able to use telemedicine applications due to visual impairment and limited health-related literacy, both common in HF [9, 10]. Therefore, oral presentation of the applications’ content using plain language might increase its usability [11].

The term avatar derives from Hinduism and means “descent”, describing the manifestation of a deity on earth [12]. Today, the term avatar is used for an animation of a person that is capable of oral communication, which makes avatar technology useful for patients with low literacy or visual impairments [13, 14]. Currently, avatars of health professionals are increasingly being used in healthcare [15]. Recent evidence suggests that the use of avatar technology can improve knowledge, self-care behavior and quality of life in chronic diseases like cancer, diabetes and depression [16–18]. Thereby, patients felt comfortable to communicate with avatars and were able to develop a strong therapeutic alliance with a virtual healthcare professional [19]. Consequently, avatar technology has the potential to facilitate the implementation of telemedicine devices in healthcare, especially for older patients who are inexperienced with technology [20]. So far, the role of avatar technology in HF care has not been studied.

This study aimed to clarify the potential role of avatar technology in telemedicine and HF care. Therefore, we integrated a nurse look-a-like avatar with the HF coach to investigate its usability and to collect information for future improvements of telemonitoring devices.

## Methods

A descriptive survey was conducted. All HF patients ≥18 years of age, diagnosed with HF, irrespective of left ventricular ejection fraction, were eligible for inclusion. Patients were randomly selected from our HF outpatient clinic in Maastricht, the Netherlands, between August 2016 and February 2017. Participation was voluntary and all patients were treated according to current European Society of Cardiology (ESC) guidelines [21]. Patients received oral and written information about the study and all gave written informed consent to participate. Patients installed the application on their smart phone or tablet computer. If necessary, the supplying company (Sananet Care B.V. Sittard, NL) took care of installation of the application. If patients did not own a smart phone or tablet computer, the company provided a tablet computer.

## Instruments

The HF coach (http://mijnhartfalencoach.nl) is an internet-based application for remote monitoring of HF patients. It regularly provides online, written questionnaires in the Dutch language based on common HF signs and symptoms to evaluate patients’ health status (Supplemental file 1). Thereby, the HF coach is based on a self-adaptive algorithm, i.e., automatically adds additional questions and adjusts the frequency of monitoring based on the presence or absence of symptoms. Educational sessions, including written information on the HF syndrome and HF care, are provided as well. Patients are able to communicate with HF nurses by sending and receiving messages and notifications via the HF coach. For this pilot study, the nurse-avatar “Molly” (Sensely, San Francisco, CA, USA; www.sensely.com) was introduced into the HF coach.

The avatar reads the written information from the health questionnaires and educational sessions provided by the HF coach out loud in the Dutch language to the patient. Voice recognition was not included in the application and patients had to check the appropriate box online to answer the questions.

All completed questionnaires were sent via a protected server to the hospital’s i‑Care desktop and evaluated by HF nurses. In cases of deterioration, patients were directly contacted by the HF nurse for further instructions.

## Data collection and follow-up

Patient characteristics and information about their digital experience were obtained after inclusion in the study during outpatient visits. The study duration was 3 months and patients received health questionnaires on a daily basis during the first month. Thereafter, questionnaires were provided once a week for 2 months, unless increasing symptoms required shorter intervals. The educational sessions could be accessed at any time. To evaluate patients’ experiences with the avatar, three different questionnaires had to be completed by the participants: the system usability scale (SUS), a self-developed patient satisfaction scale (PSS) and a general feedback form.

The SUS is a standardized, 10-item questionnaire to assess perceived usability (Supplemental file 2) [22]. Items are ranked on a scale from 1 (strongly disagree) to 5 (strongly agree) with a maximum score of 100, whereas higher scores indicate better usability [23].

Additionally, we developed a short 5‑item questionnaire to evaluate patients’ satisfaction with the avatar (PSS, Supplemental file 3). Items were ranked on a scale from 0 (not satisfied at all) to 10 (very satisfied) with a maximum score of 50.

Both questionnaires were provided after 30, 60 and 90 days of the study. After 3 months of follow-up, patients’ feedback on improving the avatar was collected based on three questions: “Do you want to talk back to Molly?” (yes/no/neutral); “Do you want Molly to leave out punctuation marks during reading?” (yes/no/neutral); “How often do you want to communicate with Molly in the future?” (daily/once a week/several times a week).

Patients were considered as completers if they completed the 3 months follow-up and at least 1 evaluation questionnaire; non-completers did not complete the 3 months follow-up and/or did not complete any evaluation questionnaire.

## Data analysis

Descriptive statistics were performed to describe baseline characteristics of the participants. To analyze differences in means of SUS and PSS over time, the Wilcoxon signed ranks test was used. A *p*-value <0.05 was considered statistically significant. All analyses were performed with IBM SPSS 25.0 (IBM, Armonk, NY, USA).

## Ethical considerations

Ethical approval for this study was obtained from the medical ethics committee of the University Hospital Maastricht (METC 16-4-013). The study conforms to the principles outlined in the Declaration of Helsinki [24].

## Results

### Patient characteristics

An older HF population (*n* = 37, mean age=67 years, standard deviation (SD) 10.9 years, age range 45–87 years, 70% male) with mild to moderate symptoms was included. In total, 26 patients (70%) completed the study. Completers were younger and more digitally experienced than non-completers. The main reason for non-completing was that patients were unable to use the application autonomously at home (*n* = 9) and two patients felt uncomfortable working with the HF coach.

In total, 581 health questionnaires provided by the HF coach were completed during the study period. The median number of completed questionnaires per patient was 23 (interquartile range, IQR 11–33). Educational sessions were followed by 19 completers (73%).

### Usability and patient satisfaction

The mean SUS was 73 (SD=24) after 1 month and 72 (SD=10) after 3 months of follow-up, which was not significantly different (*p* = 0.40) (Table 1). The mean PSS score was 42 (SD=5) after 1 month and 39 (SD=8) after 3 months, which was not significantly different (*p* = 0.10).

**Table 1:** Mean system usability scores and patient satisfaction scales

	System usability score			Patient satisfaction scale		
Time point	Mean	SD	*p*-value	Mean	SD	*p*-value
Day 30 (*n* = 26)	73	24	–	42	5	–
Day 60 (*n* = 22)	79	18	0.66	41	7	0.98
Day 90 (*n* = 19)	72	10	0.40	39	8	0.10

*p*-value for comparing mean score from day 60 or day 90 with mean score from day 30

*n* number, *SD* standard deviation

### Patient feedback

In total, 25 patients gave additional feedback on improving the avatar via the general feedback form. Of the patients five (20%) would appreciate if Molly had voice recognition and three patients (12%) would like the avatar to leave out punctuation marks during reading. Communicating with Molly once a week was preferred by most patients (40%), followed by several times a week (28%) and daily contact (8%).

## Discussion

The majority of HF patients assessed the communication with a telemedicine application via a nurse look-a-like avatar as usable and satisfactory during the study period; however, some, particularly elderly patients, were not able to use the avatar, which needs consideration for the development of new telemedicine devices.

Telemedicine enables patients with chronic diseases to live a self-determined life in their home environment, as they can be monitored remotely to prevent deterioration leading to hospitalization or even death. Nevertheless, this approach requires patients to communicate with telemedicine applications, which may be challenged with little digital experience, visual impairment and limited health-related literacy [25]. Avatar technology might help to overcome these barriers [26]. Recently, Lin et al. investigated the usability of an avatar-supported rehabilitation program for stroke survivors [27]. Perceived usability of the avatar system was poor, with a mean SUS of 28 (SD: 18.8). Discrepancy with our results may be due to differences in avatar design. The avatar animation developed by Lin et al. was relatively abstract, whereas Molly very closely resembles a human, which might have been favorably perceived by participants [28]. Also, stroke survivors may be cognitively more limited than HF patients, making it more difficult for them to use technical applications.

Both shortage of healthcare workers and increasing numbers of HF patients threaten to overburden current healthcare systems, which may be resolved by large-scale implementation of technical applications like telemedicine devices [29]. Nevertheless, this digital transformation of care requires patients to actively participate in managing their disease [30]. Patient satisfaction is positively associated with treatment adherence and therefore a key issue in future health care [31]. It has been shown that patient satisfaction with telemedicine devices supported by avatar technology may be high [32], which could be confirmed with our results. As recommended by the participants of our study, future devices should also include voice recognition of the avatar to further improve patient satisfaction.

Despite detailed information provided at the outpatient department, older HF patients with little digital experience were often unable to use the avatar autonomously at home. New eHealth applications should consider this hurdle to a substantial part of the patient population, particularly in chronic diseases such as HF with a large proportion of old patients. In addition, this is worrisome as the COVID-19 pandemic has caused an enormous shift to telemedicine and eHealth products, and older digitally inexperienced patients might be left behind [20, 33]. Therefore, we strongly recommend health policy makers to provide adequate technical support for older patients using telemedical devices to prevent age discrimination. Moreover, patient preferences regarding future devices were diverse. Therefore, a front-end device tailored to individual needs might be better perceived than the current one size fits all approach.

The patient self-care using eHealth in chronic heart failure consortium (PASSION-HF, Interreg NEW 702) is developing an integrated eHealth product (DoctorME) to improve monitoring and treatment of HF patients [30]. Based on the results of the present trial, an avatar was integrated into DoctorME to improve communication with the patient. DoctorME is currently being evaluated in a large, multicenter clinical trial (NCT04699253).

Taken together, our results support the implementation of avatar technology in telemedicine devices to improve their usability and patient satisfaction; however, randomized studies are necessary to confirm these findings. Moreover, individual capabilities and needs of patients should be considered.

## Limitations

Usability and patient satisfaction had not been evaluated for the HF coach without avatar technology. Therefore, investigating the additional benefit of introducing an avatar into an existing device is challenging. Nevertheless, the aim of the present study was a general evaluation of the usability of avatar technology in HF patients.

Overall, the number of participants in this trial was relatively low, which is common in pilot studies [34]. In addition, no formal power calculation was performed. Still, the results show that the main questions of the study could be adequately addressed.

After inclusion, 30% of participants did not complete the study, most of them not able to use the application at home. This could have been prevented by more technical support and tailoring patient education to the patients’ needs, as digitally inexperienced patients require more basal information than digital natives.

Finally, our results have been obtained several years ago. Nevertheless, the knowledge gap regarding the role of avatar technology in telemedicine applications still exists. Therefore, our results are still of value for current and future research and eHealth use in HF care.

## Conclusion

A nurse avatar integrated with a telemedicine application for remote monitoring was assessed by HF patients as usable and satisfactory. Still, not all patients were able to use it, which needs to be considered for the development of new eHealth products. In addition, prospective, ideally randomized, studies providing adequate technical support for older adults are necessary to confirm our results.

### Supplementary Information

Supplemental files 1–3
