# Supplementary material for: Integrating avatar technology into a telemedicine application in heart failure patients: A pilot study
Source: Wien Klin Wochenschr. 2023 Feb 2;135(23-24):680–4. doi: 10.1007/s00508-022-02150-8 (PMC9894666; doi:10.1007/s00508-022-02150-8)
Supplement: Supplementary file 1 — Supplemental files 1–3 [file 508_2022_2150_MOESM1_ESM.docx]

**Supplemental files**

Supplemental file 1: HF coach questionnaire

Question 1: “Are you more breathless than usually?”

• No

• Yes, a little

• Yes, a lot

Question 2: “Did you wake up tonight because of breathlessness or tickling cough?”

• No

• Yes

Question 3: “Have your legs or feet been more swollen than usually?”

• No / Don’t know

• Yes, a little

• Yes, a lot

Question 4: “Did you suffer from abdominal distention, loss of appetite, a feeling of fullness or nausea?”

• No

• Yes, a little

• Yes, a lot

Question 5: “Did you gain weight over the past 3 days?” [This question is only offered if Question 3 or Question 4 were answered with “Yes, a little” or “Yes, a lot”]

• No / Don’t know

• Yes, less than 2 kg

• Yes, more than 2 kg

Question 6: “Did you have chest pain since our last session?”

• No

• Yes, resolved

• Yes, still present

Question 7: “Did you feel more lightheaded or faint than usually?”

• No

• Yes, a little

• Yes, a lot

Question 8: “Do you feel anxious or concerned about your heart failure?” [This question is only offered if patients reported symptoms during this or the previous session, irrespective of the type of symptoms]

• No

• Yes, a little

• Yes, a lot

Question 9: “Did you struggle more than normally to perform your daily activities?” [This question is only offered if patients reported symptoms during this or the previous session, irrespective of the type of symptoms]

• No

• Yes, a little

• Yes, a lot / Couldn’t do anything

Supplemental file 2: The System Usability Scale

1. I think that I would like to use this system frequently.

2. I found the system unnecessarily complex.

3. I thought the system was easy to use.

4. I think that I would need the support of a technical person to be able to use this system.

5. I found the various functions in this system were well integrated.

6. I thought there was too much inconsistency in this system.

7. I would imagine that most people would learn to use this system very quickly.

8. I found the system very cumbersome to use.

9. I felt very confident using the system.

10. I needed to learn a lot of things before I could get going with this system.

Supplemental file 3: Patient Satisfaction Score (score 0-5)

1. Molly reads out loud

2. Molly’s voice in general

3. Clarity of Molly’s voice

4. Instructions for using Molly

5. Support with technical problems
